# Case report of a phantom pheochromocytoma

**DOI:** 10.11613/BM.2020.021003

**Published:** 2020-06-15

**Authors:** Caroline M Joyce, Audrey Melvin, Paula M O’Shea, Seán J Costelloe, Domhnall J O’Halloran

**Affiliations:** 1 Department of Clinical Biochemistry, Cork University Hospital, Ireland; 2 Department of Endocrinology, Cork University Hospital, Ireland; 3 Department of Clinical Biochemistry, Saolta University Health Care Group, Galway University Hospital, Ireland

**Keywords:** case report, pheochromocytoma, metanephrines, false positive, sulfasalazine

## Abstract

Plasma free metanephrines or urinary fractionated metanephrines are the biochemical tests of choice for the diagnosis of pheochromocytoma as they have greater sensitivity and specificity than catecholamines for pheochromocytoma detection. This case highlights the preanalytical factors which can influence metanephrine measurement and cause a false positive result. It describes a patient with a high pre-test probability of pheochromocytoma due to hypertension and a past medical history of adrenalectomy for a purported pheochromocytoma in her home country. When biochemical screening revealed grossly elevated urine normetanephrine in the presence of a previously identified right adrenal lesion, there was high clinical suspicion of a pheochromocytoma. However, functional imaging did not support this view which prompted additional testing with plasma metanephrines. Results for plasma and urine metanephrines were discordant and preanalytical drug interference was suspected. Patient medications were reviewed and sulfasalazine, an anti-inflammatory drug was identified as the most likely analytical interferent. Urinary fractionated metanephrines were re-analysed using liquid chromatography tandem mass spectrometry (LC-MS/MS) and all metanephrines were within their reference intervals. This case illustrates how method-specific analytical drug interference prompted unnecessary expensive imaging, heightened patient anxiety and resulted in lengthy investigations for what turned out to be a phantom pheochromocytoma.

## Introduction

Pheochromocytomas are rare neuroendocrine disorders derived from chromaffin cells of the adrenal medulla. Classic symptoms include paroxysmal episodes of headache, hypertension, panic attacks, sweating and tachycardia due to excess catecholamine production. It is important to diagnose pheochromocytoma early as surgery can cure the patient’s hypertension and excess catecholamine production can lead to cardiovascular complications (*1*). Diagnosis depends on biochemical evidence of inappropriate catecholamine production. Traditionally three consecutive 24-hour urine collections were performed to allow for intermittent secretion of catecholamines by pheochromocytoma (*2*). Fractionated urinary metanephrines or plasma free metanephrines are now the preferred biochemical screening test as these catecholamine metabolites are continuously produced by the tumour (*3*). Plasma metanephrines have a sensitivity of 98% and a specificity of 89% for the detection of pheochromocytoma (*4*). Clinical practice guidelines recommend the use of high performance liquid chromatography with electrochemical detection (HPLC-ECD) or liquid chromatography with tandem mass spectrometry (LC-MS/MS) for the measurement of metanephrines (*3*). However, LC-MS/MS detection has higher specificity and is considered less prone to analytical interference (*5*). Cross sectional computed tomography (CT) remains the imaging modality of choice for pheochromocytoma detection with Magnetic Resonance Imaging (MRI) reserved for cases with metastatic disease (*3*). Pheochromocytomas can occur sporadically or in association with a familial syndrome. It is imperative to diagnose pheochromocytoma early and prior to surgery to avoid a hypertensive crisis during anaesthesia.

## Case report

A 53 year old Polish lady was referred to endocrinology services for evaluation of an incidental right sided adrenal lesion on a background of a left adrenalectomy for a purported pheochromocytoma five years earlier. At assessment, medical records including the histology report on the previously excised adrenal gland were unavailable to the medical team. The patient was known to have hypothyroidism, rheumatoid arthritis and renal calculi. Despite medical management with four antihypertensive agents, the patient had uncontrolled hypertension. Clinical assessment was otherwise unremarkable. Functional characterisation of the adrenal incidentaloma was undertaken by biochemical and radiological assessment performed in accordance with clinical management guidelines (*6*). While attending her consultant endocrinologist for investigation of hypertension, the patient gave full informed consent for the performance of laboratory testing relevant to her presenting condition. Routine biochemical work-up for hypertensive patients includes a dexamethasone suppression test, aldosterone-renin ratio and urinary catecholamines/metanephrines. Furthermore, based on her past history, this patient was consented for genetic testing for pheochromocytoma. On finding grossly elevated urinary metanephrines, the patient was readmitted to hospital and consented to have testing for plasma metanephrines.

## Laboratory analyses

Urine was collected over a 24 hour (24h) period in a dark container (protected from light) containing 10 mL hydrochloric acid (HCl) as preservative to ensure pH < 3 and stored at 4°C prior to analysis (*7*). The patient was advised to empty her bladder on the morning at the start of the collection. This time was noted and the patient collected all urine passed over the following 24h period. On completion, the patient labelled the container with their name, date, and time of collection and transported it to the laboratory for analysis. Three consecutive 24h urine collections were provided with creatinine and pH measured on each to ensure a complete 24h collection and appropriate acidification (pH < 3). Urinary fractionated metanephrines were analysed by HPLC-ECD on a Waters Alliance 2690 separations module. Urine normetanephrine was grossly elevated in all 3 collections measuring 35,237, 19,440 and 5813 nmol/24h which was 2 to 12-fold the upper reference level (URL). This raised clinical suspicion that the adrenal lesion was a pheochromocytoma. The patient’s urinary catecholamine and metanephrine results are recorded in Table 1. Clinical features did not suggest glucocorticoid excess but an overnight dexamethasone suppression test (DST) was performed and a low morning cortisol (55 nmol/L) excluded subclinical Cushing’s syndrome. Aldosterone and renin were measured to investigate the patient’s hypertension but the aldosterone to renin ratio was below the cut-off value for primary hyperaldosteronism. Gut hormones and chromogranin A were also measured and were within normal reference intervals.

**Table 1:** Patient’s urinary catecholamine and metanephrine results

**Analyte, unit**	**Specimen/day 1**	**Specimen/day 2**	**Specimen/day 3**	**Reference range**
24hr Urine volume, mL	1690	1800	1680	800-2000*
Creatinine, mmol/24h	6.9	6.9	7	5.3-14.0
Noradrenaline, nmol/24h	423^†^	378^†^	655^†^	0-900
	402^‡^	370^‡^	362^‡^	82-554
Adrenaline, nmol/24h	17^†^	18^†^	84^†^	0-230
	14^‡^	12^‡^	12^‡^	8-77
Dopamine, nmol/24h	3650^†^	3366^†^	3377^†^	0-3330
	1690^‡^	3535^‡^	3344^‡^	561-3007
Normetanephrine, nmol/24h	35,237^†^	19,440^†^	5813^†^	0-2800
	2924^‡^	3978^‡^	3864^‡^	< 4900
Metanephrine, nmol/24h	17^†^	486^†^	302^†^	0-1800
	439^‡^	630^‡^	537^‡^	< 2000
Methoxytyramine, nmol/24h	NT^†^	NT^†^	NT^†^	
	2636^‡^	1638^‡^	2117^‡^	< 2550
*with a normal fluid intake of about 2 litres *per* day. h - hour. mL - millilitres. ^†^HPLC-ECD - high performance liquid chromatography with electrochemical detection. ^‡^ LC-MS/MS - liquid chromatography with tandem mass spectrometry detection. NT - not tested.				

Venous whole blood was collected into a specimen tube containing ethylenediaminetetraacetic acid (EDTA) for genetic testing and refrigerated at 4°C prior to testing. Some familial syndromes are associated with bilateral adrenal pheochromocytoma so mutation analysis was performed by Sanger sequencing for a panel of pheochromocytoma susceptibility genes; *PRKAR1A, SDHB, SDHC, SDHD, SDHAF2, TMEM127, RET, VHL and MAX*. Additionally, screening for deletion or duplication within coding exons was performed by multiplex ligation dependent probe amplification (MLPA) analysis of *VHL*, *SDHB, SDHC, SDHD and SDHAF2* genes but no pathogenic mutation was detected.

Blood for plasma metanephrines was drawn after an overnight fast with the patient resting, supine and cannulated for 30 minutes. Whole blood was collected into two EDTA specimen tubes (Greiner Bio-One, Kremsmünster Austria), placed immediately on ice, transported to the laboratory, processed within 30 min of phlebotomy and frozen at - 20°C pending analysis. Plasma metanephrines were analysed using hydrophobic interaction liquid chromatography (HILIC)-based tandem mass spectrometry (HPLC-MS/MS) and results are shown in Table 2. The patient’s plasma metanephrines were not elevated and did not support a diagnosis of pheochromocytoma.

**Table 2:** Patient’s plasma metanephrine and chromogranin A results

**Analyte, unit**	**LC-MS/MS results**	**Reference range**
Normetanephrine, pmol/L	1156	120-1180
Metanephrine, pmol/L	129	80-150
3’Methoxytyramine, pmol/L	100	< 180
Chromogranin A, pmol/L	30	0-60
LC-MS/MS - liquid chromatography with tandem mass spectrometry detection.		

## Considered diagnoses/Interventions/Further investigations

The finding of elevated urinary normetanephrines, in the context of a previously identified right adrenal lesion and past medical history of left adrenalectomy for presumed pheochromocytoma, raised the clinical suspicion of a metachronous pheochromocytoma in the right adrenal gland, which would require surgical resection. However, the normal plasma normetanephrine result (1156 pmol/L) coupled with a normal chromogranin A result (30 pmol/L) was in conflict with this diagnosis and the patient warranted more extensive radiological and biochemical investigation ahead of a surgical intervention that would have rendered her hypoadrenal. The computed tomography and MRI of the adrenals showed a right sided benign adrenal adenoma (size 4.2 cm x 1.9 cm), which had remained stable in size over a 5 year period. Further imaging with ^123^I-Metaiodobenzylguanidine single-photon emission computed tomography MIBG-SPECT revealed a non-functional lesion with no abnormal uptake of radiotracer in the right adrenal gland. A whole body octreotide scan did not reveal evidence of pentetreotide-avid disease and failed to locate a neuroendocrine tumour. Computed tomography imaging with contrast of the Thorax-Abdomen-Pelvis regions did not locate an extra-adrenal pheochromocytoma but showed a stable non-functional right sided adenoma with no features of left sided recurrence.

## What happened/Solution

In the absence of other evidence supporting a diagnosis of pheochromocytoma, there was concern about the initial elevated urinary metanephrine result, which may have been a false positive. A stored frozen aliquot of the original urine sample analysed by HPLC-ECD was reanalysed by LC-MS/MS on an API 3200 QTrap tandem mass spectrometer and the urinary metanephrine results were within the reference interval. This finding suggested that the original urine normetanephrine result was likely a false positive caused by pre-analytical drug interference and that the right adrenal lesion represented a non-functioning adrenal adenoma, as reported by MIBG scintigraphy. Patient medications recorded in table 3 were reviewed and sulfasalazine, an anti-inflammatory drug used for the patient’s rheumatoid arthritis (2000 mg daily) was considered the most likely culprit. Sulfasalazine was previously shown to cause falsely elevated normetanephrine and interfere in HPLC-ECD urine analysis (*8*). The pathology slides and tissue block from the previous left adrenalectomy on this patient were requested from her home country for external review and revealed an adrenal cortical adenoma with no evidence of malignancy.

**Table 3:** Patient’s medications during urine and plasma metanephrine measurement

**Medication**	**Dosage**
Bisoprolol	5mg OD
Doxazosin	6mg OD
Cholecalciferol	1200U OD
Levothyroxine	50mg OD
Hydrochlorothiazide/Valsartan	80/12.5mg OD
Hydroxychloroquine	200mg BD
Sulfasalazine	1000mg BD
OD - once daily. BD - twice daily	

## Discussion

Measurement of plasma free metanephrines or urinary fractionated metanephrines are the most sensitive biochemical tests for the investigation of pheochromocytoma as these o-methylated catecholamine metabolites are continuously secreted by the tumour and are not subject to episodic secretion characteristic of catecholamines (*9*). However, the low prior probability of detecting pheochromocytoma owing to its rare occurrence combined with the low diagnostic specificity of plasma metanephrines generally contributes to a high false-positive rate for biochemical screening (*4*).

Both pre-analytical and post-analytical factors can lead to false-positive or false-negative urine and plasma metanephrine results. Timing and patient posture during blood sampling is important as are diet and medications (*10*). Diet affects the measurement of dopamine and its metabolite, 3-methoxytyramine so samples should be taken after an overnight fast. Upright posture will affect sympathetic activation so drawing blood in the seated position may be associated with a 2.8 fold increase in false-positive results (*11*, *12*). Blood should be drawn in the supine position with patients recumbent for at least 30 minutes before sampling and results should be quoted with reference intervals established in the same position. Use of inappropriate reference intervals increases the likelihood of false-negative results. Borderline positive plasma metanephrine results in patients screened for pheochromocytoma should be repeated under standardised pre-analytical conditions and where possible off all potentially interfering medications (*13*). Moderately elevated results from bloods drawn in the seated position should be repeated in the supine position after 30 minutes rest (*3*). In cases with unexpected or borderline metanephrine results, the additional measurement of urinary metanephrines and chromogranin A may help to establish the diagnosis.

Sporadic pheochromocytomas may be discernible by the magnitude of the increased plasma or urinary metanephrine results above the URL. Elevations of both normetanephrine and metanephrine are rare as false-positives and should be treated as highly suspicious (*10*). Equally, elevations in a single metanephrine 3-fold higher than the URL is rare as a false-positive and should be followed up with imaging to locate a pheochromocytoma (*3*). In our case, the disparity between normetanephrine concentrations in the three consecutive 24hour urine collections, during the period of assessment was suspicious as was the lack of elevation in the parent catecholamine, noradrenaline. Our case supports the previously reported high false positive rate for urinary fractionated metanephrines (*14*).

Medications may interfere pre-analytically or analytically in the measurement of metanephrines to cause false positive results. They may increase catecholamine release (*e.g.* caffeine) or interfere in analytical measurement as reported for midodrine, methenamine and L-DOPA (*15*-*17*). They may also interfere with neuronal uptake as reported with the antidepressant drug venlafaxine (*18*). Drug interferences may give rise to mild or moderately elevated plasma or urine metanephrine results. Sulfasalazine is a prodrug which is metabolised to 5-aminosalicylic acid (mesalamine) in the gut. In a study comparing HPLC-ECD and LC-MS/MS measurement of urinary metanephrines in urine samples spiked with mesalamine, sulfasalazine or normetanephrine the authors showed that HPLC-ECD analysis may be subject to interference by a metabolite of mesalamine or a molecule released into the urine during sulfasalazine treatment (*8*).

The excess secretion of catecholamines in pheochromocytoma is associated with high morbidity and mortality so prompt diagnosis is critical. Patients with pheochromocytoma have a higher rate of major cardiovascular complications, probably due to prolonged exposure to the toxic effects of catecholamines (*19*). Some pheochromocytomas have high malignant potential, particularly those with germline mutations in the SDHB gene so genetic testing should be performed in all patients with histologically confirmed pheochromocytoma to assist with prognosis and management. Identification of a pathogenic mutation in the index case will facilitate cascade predictive testing in family members.

Despite the higher specificity and sensitivity of plasma free metanephrines, urinary fractionated metanephrines are still commonly used for pheochromocytoma screening and are recommended in clinical practice guidelines. In this case, earlier recognition of sulfasalazine as an interferent in the urinary metanephrine analysis could have prevented costly hospital admission, extensive radiological imaging and increased patient anxiety. This patient was considered for a laparoscopic right adrenalectomy based on a false positive urinary metanephrine result but following extensive radiological investigations and a comprehensive clinical review, a phantom pheochromocytoma was diagnosed.

Essential hypertension (EH) is a diagnosis of exclusion and accounts for 90-95% of all cases of hypertension. Biochemical investigations in this patient were performed to investigate a potential secondary cause because of uncontrolled hypertension despite medical management with four antihypertensive agents. Tests included: the overnight dexamethasone suppression test, aldosterone to renin ratio and urinary catecholamines/metanephrines. No identifiable cause was found and the diagnosis of essential/primary hypertension was made. The patient’s anti-hypertensive medications were reviewed and the importance of adherence to medications was explained. The patient has regular follow-up and has maintained good blood pressure control on two agents (phenoxybenzamine and bisoprolol). This case highlights the importance of taking a good drug history and critically reviewing all test results before reaching a diagnosis of pheochromocytoma.

## What YOU should/can do in your laboratory to prevent such errors

1. Request plasma free metanephrines where there is a high risk of pheochromocytoma (*e.g*. familial syndrome) or where patients are using medications that could interfere in urinary assays.
2. Take a comprehensive drug history and critically review all abnormal test results for pre-analytical interference by medications.
3. Advise clinicians to avoid medications with the potential to cause analytical interference for two weeks to one month before metanephrine analysis.
4. Comments should be added to all reports highlighting the possibility of drug interference in urine and plasma metanephrine assays. Analytical interference may be caused by labetalol, sotalol, paracetamol, methyldopa, sulfasalazine, midodrine, methenamine and buspirone. Pharmacodynamic interference may be caused by sympathomimetics (caffeine, ephedrine, amphetamine, nicotine), cocaine, tricyclic antidepressants, monoamine oxidase inhibitors and phenoxybenzamine.
